# Simulated poultry-house PM_2.5_ exposure reveals a gut–lung axis mechanism of microbial propionate in protecting against pneumonia

**DOI:** 10.1128/aem.01841-25

**Published:** 2026-01-14

**Authors:** Kai Wang, Junze Liu, Cuiguang Li, Yuan Li, Dan Shen, Chunmei Li

**Affiliations:** 1 Research Centre for Livestock Environmental Control and Smart Production, College of Animal Science and Technology, Nanjing Agricultural University 524556, Nanjing, China; Universidad de los Andes, Bogotá, Colombia

**Keywords:** PM_2.5_-induced pneumonia, gut-lung axis, microbiota–immune interaction, SCFA

## Abstract

Poultry house fine particulate matter (PM_2.5_) poses significant respiratory risks to poultry by penetrating deep into the lung and triggering inflammatory cascades. In this study, 21- to 28-day-old broilers were exposed to total suspended particulates enriched in PM_2.5_ (2 mg/m³, 2 h/day) to investigate pulmonary injury and gut–lung axis perturbations. PM_2.5_ exposure induced collapse of the hexagonal lobular architecture, elevated pulmonary expression of IL-1β, IL-2, IL-6, IL-8, and IL-10, and activated NF-κB signaling. Concurrently, cecal microbiota α-diversity increased while the community shifted toward pro-inflammatory taxa (*Alistipes*, *Rikenellaceae*) and away from SCFA-producing species (*Bacteroides uniformis*, *Parabacteroides*). Oral supplementation of *B. uniformis* restored its abundance, replenished acetate and propionate levels, and attenuated lung injury by reducing APC activation (CD40, CCL4) and Th1 polarization (T-bet, IFN-γ, IL-18), while promoting regulatory T cell markers (FoxP3). Dietary sodium propionate supplementation in feed (0.4%) similarly mitigated pulmonary inflammation and Th1 skewing, albeit without enhancing Treg responses. These findings demonstrate that PM_2.5_-induced lung damage is intricately linked to gut dysbiosis and SCFA depletion and that restoration of *B. uniformis* or its metabolite propionate can recalibrate the gut–lung axis to suppress innate and adaptive inflammatory pathways. This work highlights microbiota- and metabolite-based interventions as promising strategies to protect poultry respiratory health and performance under air-polluted conditions.

**IMPORTANCE:**

This study reveals that poultry house-derived PM_2.5_ not only causes direct lung inflammation but also perturbs the gut–lung axis by depleting beneficial SCFA-producing bacteria. The resulting gut dysbiosis amplifies respiratory immune injury, highlighting a previously underappreciated systemic effect of airborne pollutants in livestock environments. Our findings suggest that microbiota- and metabolite-targeted dietary strategies can mitigate air pollution-induced health risks in poultry. This work provides new insights into the broader ecological and agricultural consequences of PM_2.5_ exposure and supports sustainable, non-antibiotic interventions to enhance animal welfare and productivity under deteriorating air quality conditions.

## INTRODUCTION

Particulate matter (PM) in the air is a complex mixture containing bacteria, fungi, endotoxins, and other harmful substances that pose significant threats to poultry health and welfare (1). When poultry inhale pathogen-laden particulate matter, the ciliary function of their respiratory mucosa is impaired, weakening the respiratory defense barrier (2). Among particulate matter, fine particles with diameters less than 2.5 µm (PM_2.5_) are particularly concerning due to their ability to penetrate deeply into the alveoli and carry substantial microbial and endotoxin loads. Exposure to PM_2.5_ has been shown to exert cardiotoxic effects on poultry embryos and newly hatched chicks (3, 4). Furthermore, systemic exposure to PM_2.5_ decreases average daily gain in broilers, significantly elevates pulmonary inflammatory cytokines, causes lung tissue damage, and alters lung microbiota and metabolite profiles (5). Endotoxins within the dust can reduce B cell populations in laying hens, impairing immunity and increasing disease susceptibility (6). Exposure of broilers to dust and its main components such as lipopolysaccharide (LPS) and β-glucan activates humoral immune responses, elevating blood IgM and IgG levels while inhibiting growth (7). These findings demonstrate that the size, composition, and microbial constituents of particulate matter directly impact poultry health and growth performance. In addition to poultry, high concentrations of airborne particulates in chicken houses pose serious health risks to farm workers. Long-term exposure to elevated PM levels correlates with respiratory symptoms including chronic cough, bronchitis, and impaired lung function among poultry workers (8, 9). PM_10_ and PM_2.5_ are key indicators affecting respiratory health, and chronic exposure is linked to chronic obstructive pulmonary disease, cardiovascular diseases, and lung cancer. Despite ample evidence of these occupational hazards, awareness and management of particulate exposure risks in poultry farming remain inadequate (10). Thus, monitoring and controlling PM_10_ and PM_2.5_ concentrations in poultry facilities is critical to safeguarding both worker and poultry health.

The lung, as the second largest organ by surface area, is essential for gas exchange and rich in immune cells including alveolar macrophages (AMs), alveolar epithelial cells, dendritic cells (DCs), and lymphocytes, which collectively form a robust respiratory immune barrier (11). PM_2.5_ particles reaching the alveoli interact directly with these immune cells, triggering inflammatory responses. AMs act as the first line of defense by phagocytosing foreign particles and pathogens and presenting antigens via major histocompatibility complex class II (MHC II) and costimulatory molecules such as CD40 and CD86 to activate adaptive T cell immunity (12, 13). PM_2.5_ exposure promotes pro-inflammatory M1 macrophage polarization while inhibiting anti-inflammatory M2 polarization, disrupting immune balance and inducing chronic inflammation (14, 15). Additionally, PM_2.5_ activates pulmonary dendritic cells, which migrate to lung-associated lymph nodes to stimulate specific T cell responses, further modulating immune reactions (16, 17). The T cell subsets (Th1, Th2, Th17, and regulatory T cells) play critical roles in PM_2.5_-induced immune inflammation through transcription factors such as T-bet and GATA3 that regulate their differentiation and balance, thereby influencing lung inflammation progression (18, 19).

The gut-lung axis refers to the bidirectional communication between the gastrointestinal and respiratory systems, mediated by microbiota, immune responses, and metabolites (20). Gut microbes produce metabolites such as short-chain fatty acids that influence lung immunity by promoting anti-inflammatory effects, while lung inflammation or infection can disrupt gut microbiota balance, leading to systemic immune dysregulation. This interplay is well documented in human respiratory diseases like asthma and COPD, where gut microbial alterations correlate with lung pathology and gut-targeted treatments have shown benefits (21). Although less explored in poultry, this axis likely plays a crucial role in broiler health, particularly under PM_2.5_ exposure which induces lung inflammation and microbiota disturbance. Understanding how PM_2.5_ impacts both pulmonary and intestinal microbiota is essential for comprehensively addressing the systemic effects of air pollution, thereby informing strategies to enhance respiratory and gut health in poultry production.

In summary, particulate matter—especially PM_2.5_—is ubiquitous in poultry farming environments and poses significant risks to poultry respiratory health, growth performance, and farm worker well-being. Understanding the immune responses of lung-resident cells and the regulation of the gut-lung axis is key to elucidating the mechanisms of PM_2.5_-induced inflammation. Nonetheless, systematic studies on the interactions between pulmonary inflammation and intestinal microbiota in broilers exposed to PM_2.5_ are lacking. This study aims to explore the effects of PM_2.5_ exposure on pulmonary inflammation and gut microbial ecology in broilers, providing a theoretical foundation and practical guidance for respiratory health management and environmental control in poultry production.

## MATERIALS AND METHODS

### Animal maintenance

#### Trial 1

Twenty-four 21-day-old male AA broilers were randomly divided into two groups (*n* = 12 per group): control (CON, unexposed) and PM-exposed (PM). Exposure was performed with exposure devices under consistent environmental conditions (temperature, humidity, ventilation, lighting, and stocking density). In the PM group, broilers were exposed to total suspended particles (TSP) containing PM_2.5_ at a concentration of 2 mg/m³ for 2 h daily (8:30–10:30 AM) over 7 consecutive days (day 21–28, Fig. S1A). The CON group received filtered air. This whole-body exposure setup simulated typical poultry house conditions. Outside exposure periods, broilers were housed in a clean facility with background PM_2.5_ levels of 0.526 ± 0.011 mg/m³. PM_2.5_ was introduced into the chambers using a BT901 dry aerosol generator (Bite, China), connected via tubing and driven by a pump. Real-time concentrations of airborne particles were recorded every 2 min using a JK90-FC-M3 aerosol spectrometer (JSA, China). The CON chambers were supplied with particle-free indoor air, and the PM chamber’s dose was adjusted based on real-time measurements. Particle concentration data for both groups and time-course distributions during exposure are shown in Fig. S2A. After each exposure, chambers were thoroughly washed with water and disinfected under UV light for 16 h. The aerosol generator tubing and injection ports were cleaned with water and 75% ethanol and then soaked in ethanol until the next use.

#### Trial 2

Twenty-seven 14-day-old male AA broilers were randomly assigned to three groups (*n* = 9 per group): CON (control, unexposed), PM (PM_2.5_ exposure), and PM+B.U (PM exposure + *B. uniformis* gavage). From day 14 to day 28, broilers in the PM+B.U group received a daily oral gavage of 1 mL *B. uniformis s*uspension (10⁹ CFU/mL), while birds in the CON and PM groups were gavaged with 1 mL of sterile culture medium. From day 21 to day 28, broilers in the PM and PM+B.U groups were exposed to PM_2.5_ at 2 mg/m³ for 2 h per day in an exposure chamber, simulating poultry house conditions (Fig. S1B). The exposure was conducted in exposure devices under controlled environmental conditions (temperature, humidity, ventilation, lighting, and density). Birds in the PM and PM+B.U groups shared one chamber with 18 independent exposure devices, while the CON group of the same age and similar body weight was housed in the other chamber. The exposure chambers were located in a separate room with a background PM_2.5_ level of approximately 0.153 ± 0.002 mg/m³. PM_2.5_ concentrations were monitored in real time and adjusted accordingly to maintain target exposure levels. All birds were euthanized on day 28, and samples were collected from each group (*n* = 9). Details of the particle size distributions and time-course distribution in each group are presented in Fig. S2B.

#### Trial 3

Forty-five 14-day-old male AA broilers were randomly assigned to five groups (*n* = 9): CON (unexposed control), PM (PM_2.5_ exposure), PM+0.1%SP, PM+0.2%SP, and PM+0.4%SP (PM_2.5_ exposure with 0.1%, 0.2%, or 0.4% sodium propionate supplementation in feed, respectively). Sodium propionate was administered via feed from days 14 to 28. From days 21 to 28, broilers in all PM-exposed groups were subjected to daily PM_2.5_ exposure (2 mg/m³, 2 h/day) in two identical chambers with eighteen exposure devices under controlled environmental conditions (Fig. S1C). Eighteen PM_2.5_ exposure devices were located in a separate room with a background PM_2.5_ level of 0.148 ± 0.001 mg/m³. Real-time monitoring ensured stable PM_2.5_ concentrations. All birds were euthanized on day 28 for sample collection. Particle concentration data and time-course distributions during exposure are shown in Fig. S2C.

### Sampling

At the end of each trial, body weight and feed intake were recorded throughout the exposure period. After 7 days of exposure, broilers were fasted for 12 h and then euthanized by cervical dislocation under ether anesthesia. From each bird, a 0.5 cm × 0.5 cm × 0.5 cm tissue sample was collected from the center of the left lung and fixed in 4% paraformaldehyde for H&E staining. A 2-cm segment of jejunum was also collected, rinsed with saline, and fixed for histological analysis when applicable. A central right lung tissue sample and, where appropriate, cecal contents or jejunal tissue were rapidly frozen in liquid nitrogen and stored at –80°C for subsequent molecular and biochemical analyses.

### Histological evaluation of lung injury

Lung tissue samples were fixed in 4% paraformaldehyde for 24 h, dehydrated, cleared, embedded in paraffin, and sectioned into 5 μm slices for hematoxylin and eosin (H&E) staining. Lung injury was assessed blindly under a light microscope. At 200× magnification, four random fields per section were examined for hemorrhage, inflammatory cell infiltration, and capillary structural integrity. At 20× magnification, four additional fields (each containing at least one tertiary bronchus) were evaluated for alveolar septal thickness, alveolar structure integrity, and lobular hexagonal architecture. Each parameter was scored on a scale of 0 to 4 (0 = no injury; 4 = maximal injury), and the average score across all six parameters was defined as the lung injury score (22).

### RNA extraction and quantitative real-time PCR

Total RNA was extracted from lung tissue using an RNA extraction kit (R401-01, Vazyme, China), and the concentration and purity of RNA were assessed using a NanoDrop spectrophotometer. First-strand cDNA was synthesized from 1 μg of total RNA using a reverse transcription kit (RK20429, ABclonal Technology, China) following the manufacturer’s instructions. Quantitative real-time PCR (qPCR) was performed on an ABI QuantStudio 7 system using Genious 2× SYBR Green Fast qPCR Mix (RK21204, ABclonal Technology, China). The qPCR cycling conditions were as follows: initial denaturation at 95°C for 3 min, followed by 40 cycles of 95°C for 10 s and 60°C for 30 s. Melt curve analysis was conducted to confirm the specificity of amplification. Each sample was run in triplicate, and no-template controls were included in each assay. Relative mRNA expression levels were calculated using the 2^–ΔΔCT^ method, with β-actin used as the endogenous reference gene due to its stable expression across treatments

### 16S rDNA sequencing and microbiota analysis

A total of 5 samples per group (*n* = 5) were collected for 16S rDNA sequencing. Total DNA was extracted using the HiPure Stool DNA Kit (D3141, Magen Biotechnology, China). The V3–V4 hypervariable regions of the bacterial 16S rRNA gene were amplified using universal primers (Forward: 5′-CCTACGGGNGGCWGCAG-3′; Reverse: 5′-GGACTACHVGGGTATCTAAT-3′), yielding ~466 bp amplicons. Q5 High-Fidelity DNA Polymerase (M0491, New England Biolabs, USA) was used for amplification. The first-round PCR conditions were: 95°C for 5 min, followed by 30 cycles of 95°C for 1 min, 60°C for 1 min, 72°C for 1 min, and a final extension at 72°C for 7 min. A second-round PCR was performed to add sequencing adapters under the same thermal conditions but with 12 cycles. Amplicons were purified, quantified using the ABI StepOnePlus Real-Time PCR System (Life Technologies, USA), and pooled in equimolar concentrations. Barcoded libraries (6–8 bp, allowing one mismatch) were demultiplexed using custom scripts and sequenced on an Illumina NovaSeq 6000 platform (Illumina, USA) with paired-end 250 bp reads (PE250). Raw data were processed by Gene Denovo Biotechnology Co. (Guangzhou, China), including quality control, trimming of primers and adapters, and removal of low-quality reads. DADA2 (v1.14) in R was used to infer non-chimeric amplicon sequence variants (ASVs), and taxonomic classification was performed using the SILVA 138 database. Bioinformatics analysis was conducted on the Omicsmart platform (http://www.omicsmart.com), an online tool for real-time and interactive microbiome data analysis.

### Isolation and cultivation of *Bacteroides* spp.

*Bacteroides* strains were isolated using KVLB selective medium supplemented with 20 mg/200 mL kanamycin and 1.5 mg/200 mL vancomycin. Fresh cecal contents were collected from AA broilers and placed in sterile centrifuge tubes. Samples were serially diluted in sterile phosphate-buffered saline (PBS) at a ratio of 1:9. Dilution gradients of 10⁻⁷, 10⁻⁸, and 10⁻⁹ were selected, and 100 μL of each dilution was spread onto KVLB agar plates. Plates were incubated anaerobically at 37°C for 24 h. Colonies with diameters of 1–2 mm were selected and streaked repeatedly until single, contaminant-free colonies were obtained. Pure isolates were sent to Sangon Biotech (Shanghai, China) for Sanger sequencing. The resulting sequences were compared against the NCBI database for species identification. *B. uniformis* was cultured in ATCC Medium 1490 under anaerobic conditions at 37°C for 24 h using anaerobic culture pouches. The bacterial suspension was serially diluted, and 100 μL of appropriate dilutions was plated onto Columbia blood agar. After 24 h of anaerobic incubation at 37°C, colony-forming units (CFU) were counted to determine the viable bacterial concentration. Bacterial suspensions were then diluted to the required concentrations, aliquoted into sterile tubes, and stored at 4°C for subsequent use.

### Inflammatory cytokine measurement

Lung tissues were homogenized using a Tiss-24QL biological sample homogenizer. During homogenization, sample tubes were kept in a metal adapter to maintain a low temperature. The homogenate supernatant was collected after centrifugation at 3000 rpm for 20 min at 4°C and used for inflammatory cytokine assays. Levels of IFN-γ, IL-12, and IL-18 were measured using chicken-specific ELISA kits supplied by Nanjing Jiancheng Bioengineering Institute (Nanjing, China).

### Short-chain fatty acid analysis

A 25% metaphosphoric acid solution was prepared by dissolving 25 g of metaphosphoric acid in 100 mL of double-distilled water. Then, 0.6464 g of crotonic acid was added to 100 mL of this solution to prepare the metaphosphoric acid–crotonic acid solution. Chromatographic-grade acetic acid (0.91 g), propionic acid (0.37 g), butyric acid (0.1765 g), valeric acid (0.1985 g), isobutyric acid (0.1765 g), and isovaleric acid (0.1985 g) were each dissolved in double-distilled water and diluted to 100 mL as standards. For sample preparation, 0.1 g of cecal content was mixed with 0.7 mL double-distilled water, followed by the addition of 0.2 mL metaphosphoric acid–crotonic acid solution, and stored at −20°C overnight. After thawing, samples were centrifuged twice at 12,000 rpm for 10 min, and the supernatant was filtered through a 0.22 μm syringe filter before injecting 0.6 μL into the chromatograph. Retention times for each short-chain fatty acid were determined using standard solutions mixed with the metaphosphoric acid–crotonic acid solution. Concentrations of short-chain fatty acids in the samples were calculated using correction factors derived from the peak areas and concentrations of the standards and internal crotonic acid, following the formula:

Concentration (mM) = (Sample peak area × Internal standard peak area × Standard concentration)/(Sample internal standard peak area × Standard peak area).

### Statistical analysis

All data were analyzed using GraphPad Prism version 9.20. One-way ANOVA was performed to compare data between groups, followed by Tukey’s multiple comparisons test. Pearson correlation analysis was used to assess the relationship between cecal short-chain fatty acid (SCFA) concentrations and pulmonary inflammation indicators. Data are presented as mean ± standard error of the mean (SEM). A *P*-value of < 0.05 was considered statistically significant, and *P* < 0.01 was considered highly significant.

## RESULTS

### PM exposure in chicken coop on lung tissue morphology and lung injury score of broiler chickens

**Fig 1:**
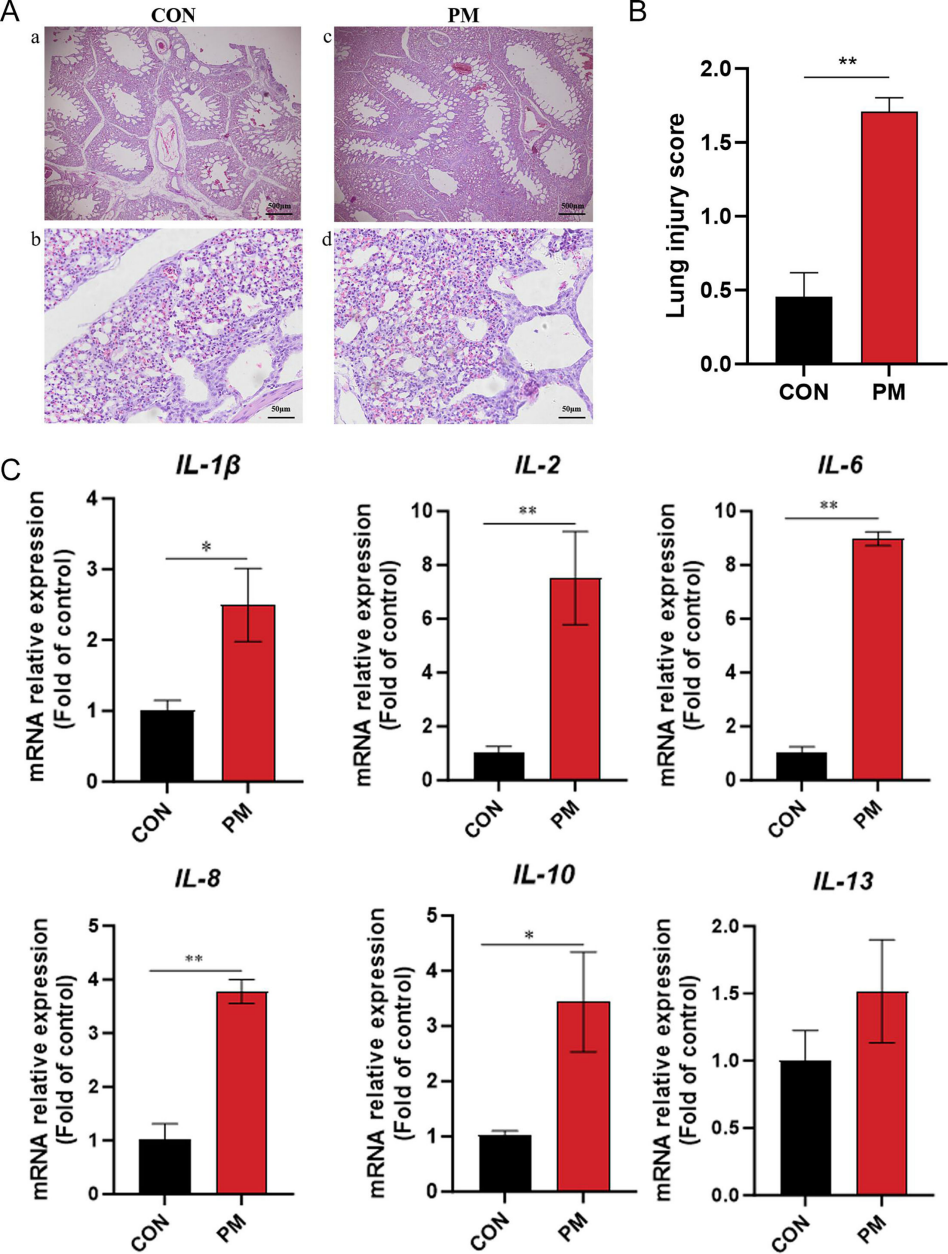
PM exposure on lung morphology and injury in broilers. (**A**: a–d) Representative H&E-stained lung tissue sections from control (CON) and PM2.5-exposed (PM) broilers after 7 days (scale bars: 100 µm for 20× magnification, 20 µm for 200× magnification). PM exposure disrupted the hexagonal structure of pulmonary lobules and caused alveolar septal thinning or loss, along with increased inflammatory cell infiltration. (**B**) Quantitative lung injury scores showing significantly higher damage in the PM group compared to CON (*P* < 0.01). (**C**) Inflammatory factors mRNA abundance. Data are presented as mean ± SEM, *n* = 6 per group. * indicates *P* < 0.05 and ** indicates *P* < 0.01.

After a 7-day exposure to PM, lung tissues were collected from broilers and subjected to hematoxylin-eosin (H&E) staining for histopathological analysis. As shown in Fig. 1A, under 20× magnification, the hexagonal structure of pulmonary lobules in the PM group was disrupted, with thinning or even disappearance of the alveolar septa. Under 200× magnification, increased infiltration of inflammatory cells was observed in the lung tissues of the PM group. Lung injury scores of all samples indicated that the PM group exhibited significantly higher scores compared to the CON group (*P* < 0.01, Fig. 1B), suggesting that PM exposure caused severe pulmonary damage in broilers.

To evaluate the effects of PM exposure on broiler lung tissue at the genetic level, the mRNA expression of inflammatory cytokines in lung tissues was quantified. The results showed that, compared to the CON group, the PM group exhibited significantly increased mRNA expression levels of *IL-1β* and *IL-10* (*P* < 0.05, Fig. 1C) and highly significant upregulation of *IL-2, IL-6*, and *IL-8* (*P* < 0.01, Fig. 1C). No significant difference was observed in the mRNA expression of *IL-13* between the two groups.

### PM exposure in poultry house on the diversity of cecal microbiota in broiler chickens

**Fig 2:**
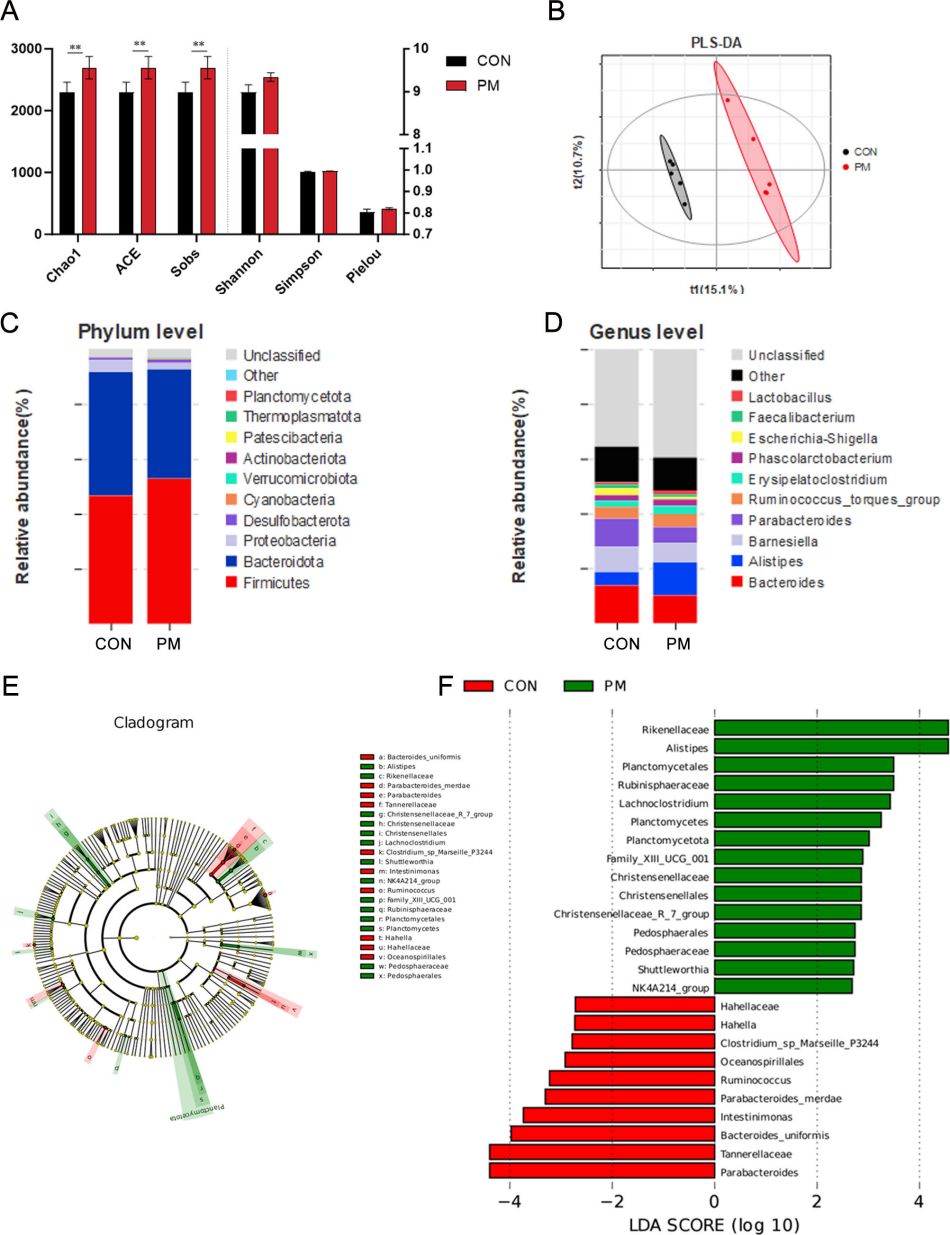
PM exposure on cecal microbiota diversity and composition in broilers. (**A**) Alpha diversity indices (Sobs, ACE, Chao1) were significantly increased in the PM group compared to CON (*P* < 0.05). (**B**) Beta diversity analysis (PCoA) showing clear separation between CON and PM groups. (**C**) Relative abundance of major bacterial phyla in cecal contents; PM group showed increased *Actinobacteriota* and decreased *Bacteroidetes* and *Proteobacteria*. (**D**) Linear discriminant analysis (LDA) identified key genera contributing to group differences; *Parabacteroides* and *Bacteroides* were enriched in CON, while *Alistipes* was elevated in PM. (**E, F**) LDA scores of differential taxa with values > 4 shown. *n* = 5. ** indicates significant differences, *P* < 0.01.

Rarefaction curves were constructed to evaluate the sequencing depth and species richness of the microbial communities (Fig. S3). The curves for all samples tended to level off, indicating that the sequencing depth was sufficient and had captured most of the microbial diversity present in each sample. Comparison of the cecal microbial communities between the two groups revealed that PM_2.5_ exposure significantly increased the α-diversity indices, including Sobs, ACE, and Chao1, in the cecal contents of broilers (*P* < 0.05; Fig. 2A). β-Diversity analysis showed a clear separation between the two groups (Fig. 2B). At the phylum level, Bacteroidetes and Firmicutes were the dominant phyla in the cecal microbiota. Compared to the CON group, the PM group showed an increased relative abundance of *Actinobacteriota*, and decreased abundances of *Bacteroidetes* and *Proteobacteria* (Fig. 2C). At the genus level, *Bacteroides* and *Alistipes* were predominant in both groups. Notably, *Alistipes* abundance was significantly higher in the PM group than in the CON group (*P* < 0.05), while *Parabacteroides* and *Bacteroides* abundances were significantly reduced (*P* < 0.05). *Parabacteroides* and *Bacteroides* contributed most to the differences observed in the CON group, with linear discriminant analysis (LDA) scores greater than 4 (Fig. 2D and E).

### *B. uniformis* on lung tissue morphology in broiler chickens exposed to PM

**Fig 3:**
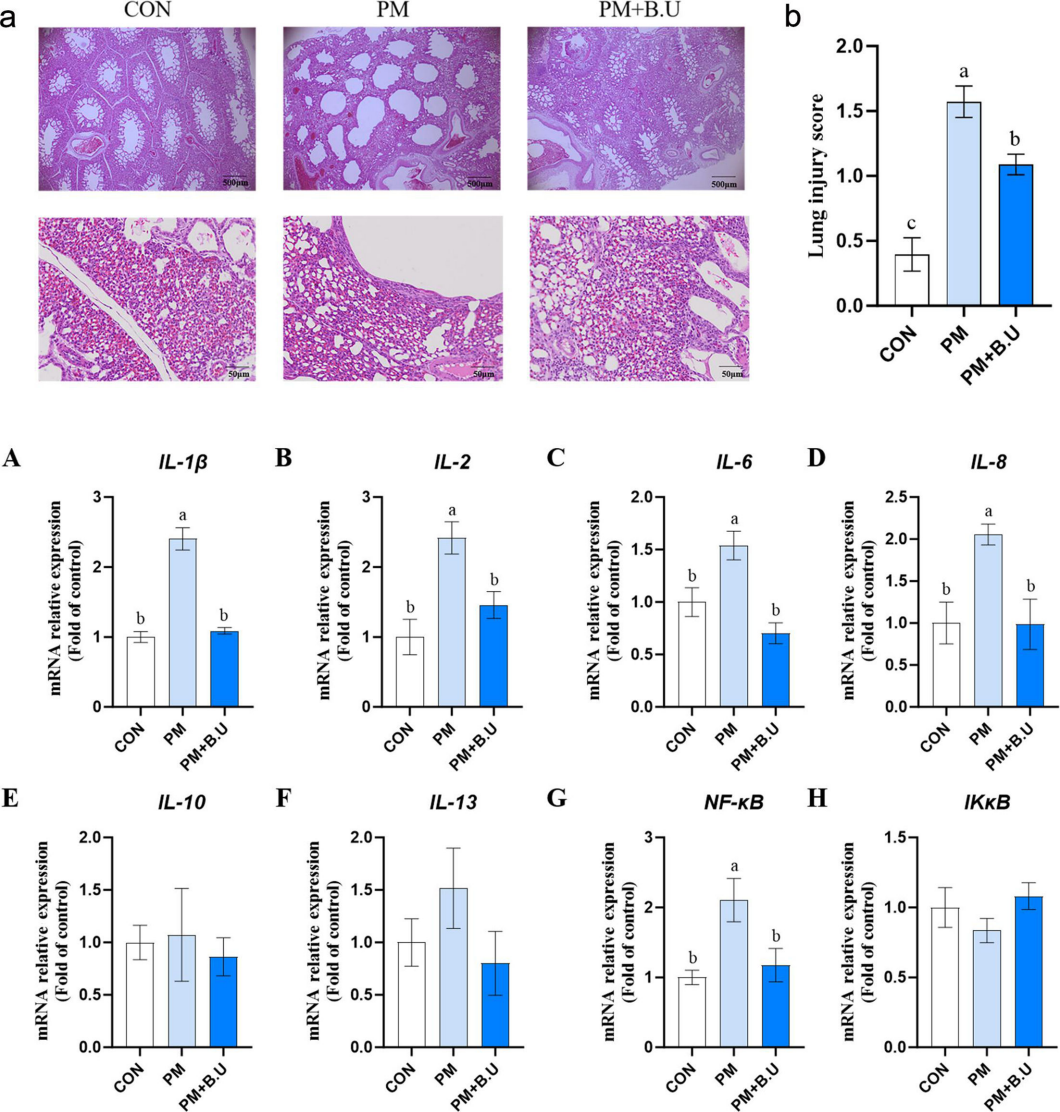
*B. uniformis* (B.U) on lung histopathology and inflammatory gene expression in PM exposed broilers. (**a**) H&E staining of lung sections from CON, PM, and PM+B.U groups showing partial restoration of alveolar septa and lobular structure in the PM+B.U group (scale bars: 100 µm). (**b**) Lung injury scores showing significant alleviation of damage in PM+B.U vs PM (*P* < 0.05). (**A–H**) Relative mRNA expression levels of pro-inflammatory cytokines *IL-1β, IL-2, IL-6, IL-8, IL-10, IL-13, NF-κb* and *IKκB* in lung tissues. Data are mean ± SEM, *n* = 8. Different lowercase letters indicate significant differences, *P* < 0.05.

After 7 days of exposure, histological analysis of broiler lung tissues was performed using H&E staining (Fig. 3A). Compared with the CON group, the PM group showed alveolar septal disruption, incomplete hexagonal structure of pulmonary lobules, and increased infiltration of inflammatory cells, whereas these pathological changes were partially alleviated in the PM+*B*.U group. Lung injury scoring revealed that the PM group exhibited significantly higher scores than the CON group (*P* < 0.05). Although the PM+*B*.U group also showed significantly higher scores than the CON group, the scores were significantly lower than those of the PM group (*P* < 0.05; Fig. 3B). Compared with the CON group, the PM group showed significantly increased mRNA expression levels of *IL-1β, IL-2, IL-6*, and *IL-8* in lung tissues (*P* < 0.05; Fig. 3C through F). However, compared with the PM group, the PM+*B*.U group exhibited significantly reduced expression of these cytokines (*P* < 0.05; Fig. 3C through F). In addition, the mRNA expression of *NF-κB* in lung tissues was significantly upregulated in the PM group compared with the CON group (*P* < 0.05; Fig. 3G).

### *B. uniformis* on pulmonary immune function in broilers exposed to PM

**Fig 4:**
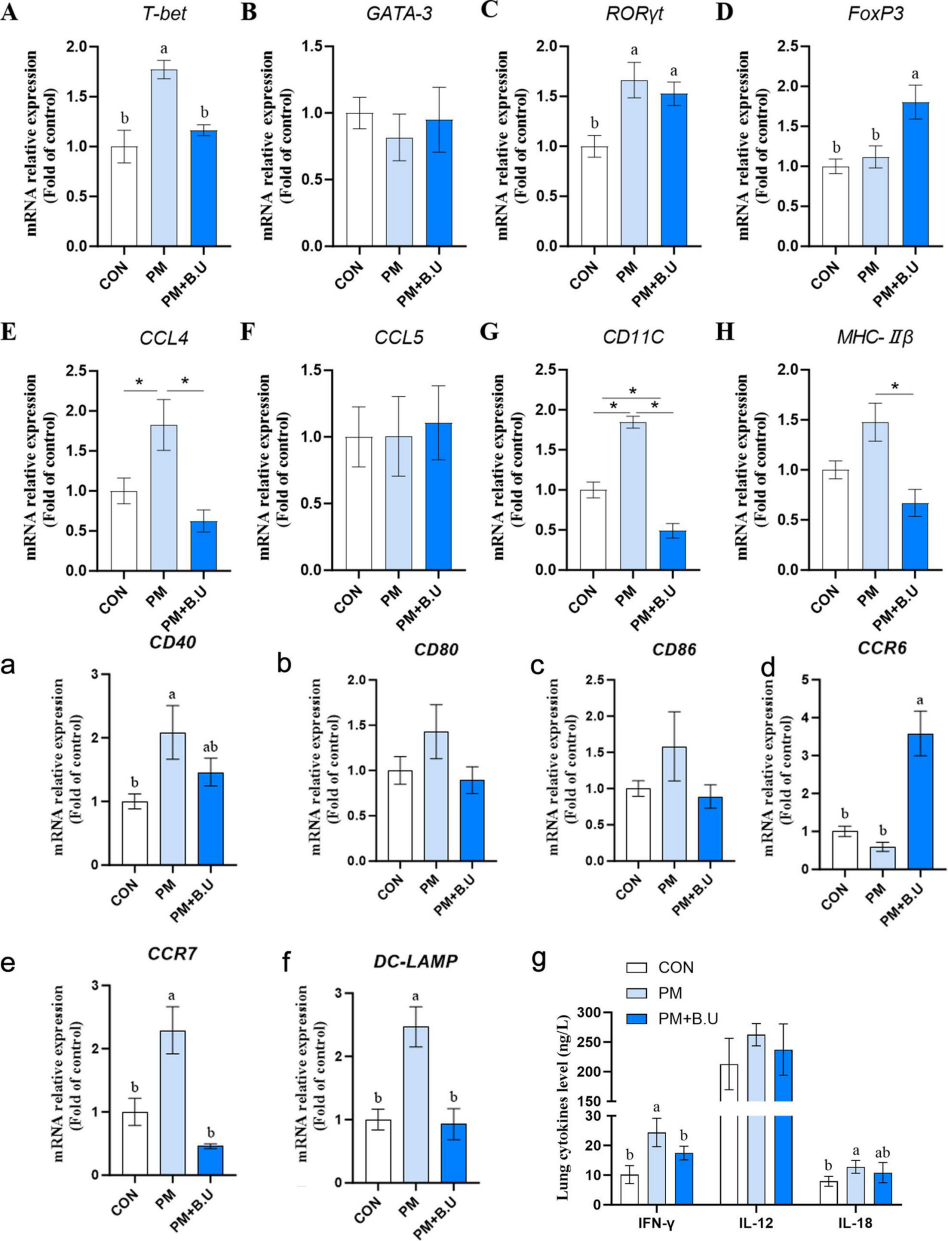
Regulation of T cell differentiation and dendritic cell activation marker genes by *B. uniformis* in lungs of PM-exposed broilers. (**A–H**) Relative mRNA levels of T cell transcription factors *T-bet, GATA3, RORγt,* and *Foxp3*, expression of APC cell activity chemokines (*CCL4, CCl5, CD11C*), and antigen presentation molecules (*MHC-IIβ*); (**a–f**) Dendritic cell activation marker genes. Relative mRNA levels of *CD40, CD80, CD86, CCR6, CCR7, DC-LAMP* expression. (**g**) Protein abundance of Th1 cytokines IFN-γ and IL-18 in lung. Data represent mean ± SEM, *n* = 8. Different lowercase letters indicate significant differences, *P* < 0.05. * indicates significant differences, *P* < 0.05.

Quantitative analysis of T cell differentiation marker genes revealed that the mRNA expression levels of *T-bet* and *RORγt* in broiler lung tissues were significantly upregulated in the PM group compared to the CON group (*P* < 0.05; Fig. 4A and C). In contrast, the PM+*B*.U group exhibited significantly lower *T-bet* mRNA expression than the PM group (*P* < 0.05), with no significant difference compared to the CON group. The *RORγt* mRNA level in the PM+*B*.U group remained significantly higher than in the CON group (*P* < 0.05). Compared to the CON group, the mRNA expression levels of *CCL4* and *CD11C* in lung tissues were significantly increased in the PM group (*P* < 0.05; Fig. 4E and G), whereas no significant changes were observed in the PM+*B*.U group. Notably, *MHC-IIβ* mRNA expression in the PM+*B*.U group was significantly reduced compared to the PM group (*P* < 0.05; Fig. 4H).

Given the observed alterations in APC function, the activation and maturation-related genes of dendritic cells (DCs) were further examined. The PM group showed significantly elevated *CD40* mRNA levels in lung tissues compared to the CON group (*P* < 0.05; Fig. 4A), while no significant differences were found between the PM+*B*.U group and the other two groups. Interestingly, the *CCR6* mRNA expression in the PM+*B*.U group was significantly higher than in both the CON and PM groups (*P* < 0.05; Fig. 4D). Expression levels of *CCR7* and *DC-LAMP* were significantly upregulated in the PM group compared to both the CON and PM+*B*.U groups (*P* < 0.05; Fig. 4E), with no significant difference observed between the CON and PM+*B*.U groups.

As T cell differentiation was affected and the Th1-specific transcription factor T-bet was regulated by *B. uniformis*, the protein levels of Th1-type inflammatory cytokines in lung tissues were subsequently quantified. The PM group showed significantly elevated levels of *IFN-γ* and *IL-18* compared to the CON group (*P* < 0.05, Fig. 4g). In the PM+*B*.U group, *IFN-γ* levels were significantly higher than in the CON group (*P* < 0.05), but significantly lower than those in the PM group (*P* < 0.05).

### *B. uniformis* on the jejunum of broiler chickens exposed to PM

**Fig 5:**
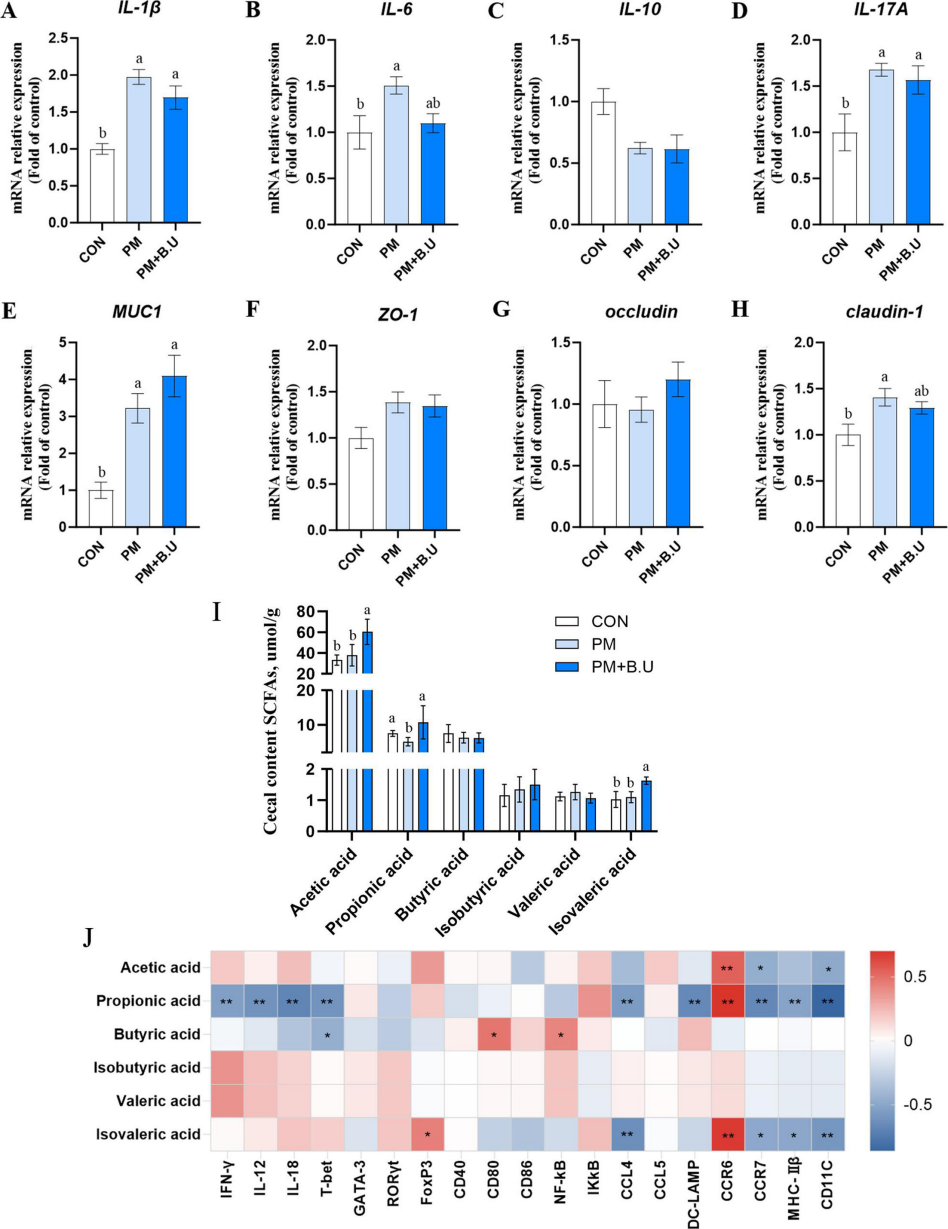
Jejunal inflammatory cytokines, barrier gene expression, and cecal SCFA analysis. (**A**) *IL-1β* mRNA expression. (**B**) *IL-6* mRNA expression. (**C**) *IL-10.* (**D**) *IL-17A* mRNA expression. (**E**) *MUC*1 mRNA expression. (**F**) *ZO-1.* (**G**) *Occludin.* (**H**) *Claudin-1* mRNA expression. (**I**) Gas chromatography analysis of cecal SCFAs. (**J**) Correlation analysis between SCFAs and lung gene expression. *n* = 8. Different lowercase letters indicate significant differences, * indicates *P* < 0.05 and ** indicates *P* < 0.01.

The relative mRNA expression levels of inflammatory cytokines and barrier function-related genes in the jejunum of broilers were analyzed. Compared to the CON group, the PM group exhibited significantly increased mRNA expression of *IL-1β, IL-6*, and IL-17A in the jejunum (*P* < 0.05; Fig. 5A, B and D). In the PM+*B*.U group*, IL-1β* and *IL-17A* expression levels remained significantly higher than those in the CON group (*P* < 0.05), while *IL-6* expression showed no significant difference among the three groups (*P* > 0.05). The expression of *MUC1* mRNA in both the PM and PM+*B*.U groups was significantly elevated compared to the CON group (*P* < 0.05; Fig. 5E). *Claudin-1* expression was significantly upregulated in the PM group relative to the CON group (*P* < 0.05; Fig. 5H), whereas no significant difference was observed between the PM+*B*.U group and the other two groups.

Short-chain fatty acids (SCFAs) in the cecal contents were analyzed via gas chromatography (Fig. 5I). The concentrations of acetate and isovalerate in the PM+*B*.U group were significantly higher than those in the CON group (*P* < 0.05), while there were no significant differences between the CON and PM groups. Propionate concentration was significantly lower in the PM group compared to the CON group (*P* < 0.05) and significantly higher in the PM+*B*.U group than in the PM group (*P* < 0.05), with no significant difference between the PM+*B*.U and CON groups. No significant differences in butyrate, isobutyrate, and valerate concentrations were detected among the groups (*P* > 0.05).

### Correlation analysis between lung tissue inflammation indicators and cecal short-chain fatty acids

Correlation analysis revealed that the concentration of acetate in cecal contents was positively correlated with pulmonary *CCR6* expression (*P* < 0.01; Fig. 5J) and negatively correlated with *CCR7* and *CD11C* expression (*P* < 0.05). Propionate concentration showed significant negative correlations with Th1-type cytokines *IFN-γ, IL-12*, and *IL-18* (*P* < 0.01), as well as with DC function-related genes *CCL4, DC-LAMP, CCR7, MHC-IIβ,* and *CD11C* (*P* < 0.01). In contrast, propionate was positively correlated with *CCR6* expression (*P* < 0.01). Butyrate concentration was significantly negatively correlated with pulmonary T-bet expression (*P* < 0.05) and positively correlated with *CD80* and *NF-κB* expression (*P* < 0.05). Isovalerate concentration was positively correlated with *FoxP3* and *CCR6* expression (*P* < 0.05) and negatively correlated with C*CL4, CCR7, MHC-IIβ,* and *CD11C* expression (*P* < 0.05).

### Sodium propionate to the diet on inflammatory in lung tissue of broiler chickens exposed to PM

**Fig 6:**
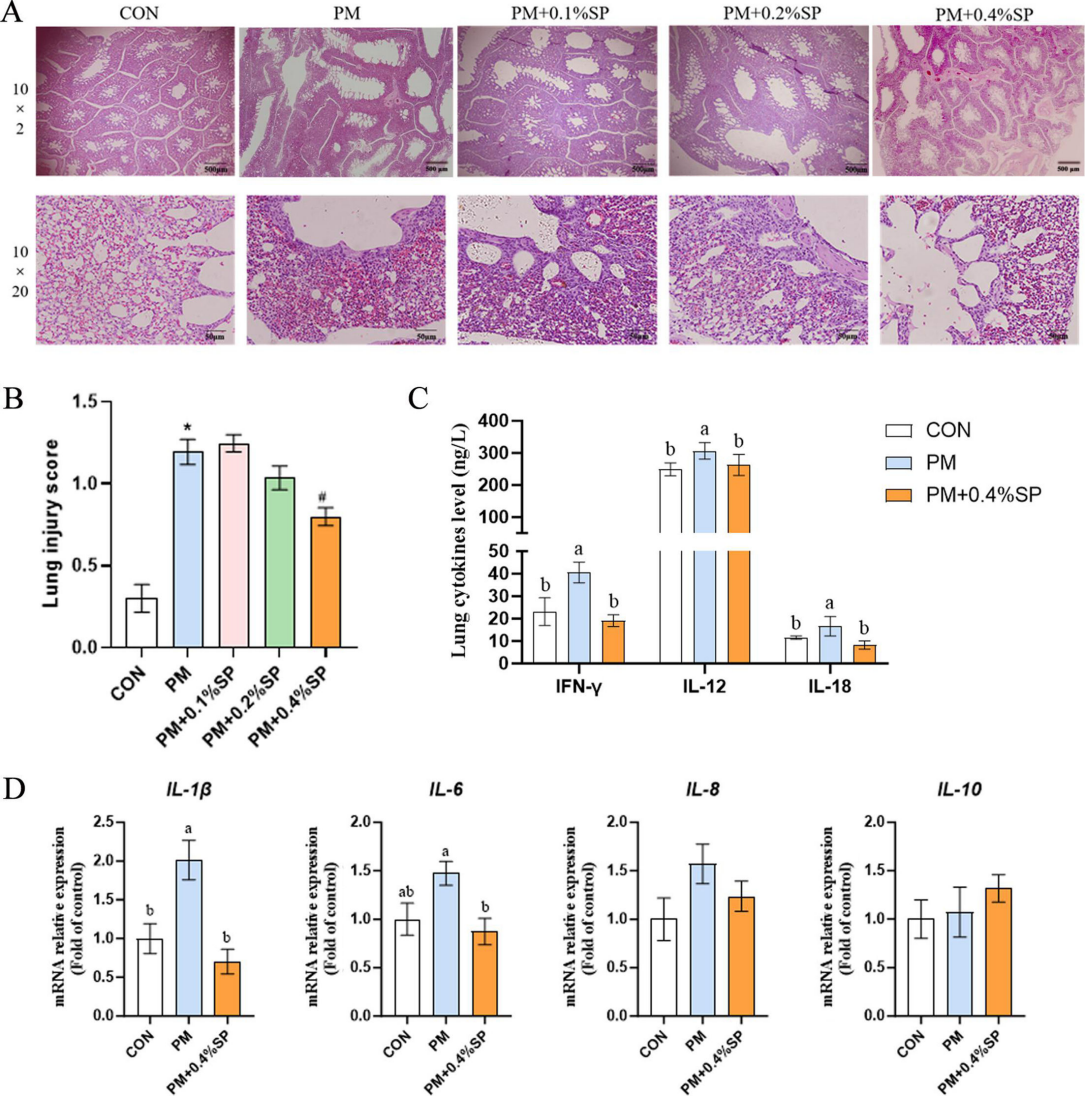
SP supplementation on lung morphology and inflammation in PM-exposed broilers. (**A**) Lung tissue H&E staining with different SP doses. (**B**) Lung injury scores. (**C**) Protein levels of IFN-γ, IL-12, and IL-18. (**D**) mRNA expression of *IL-1β, IL-6, IL-8, IL-10*. *n* = 8. Representing the maximum damage; * represents *P* < 0.05 vs CON group; # represents *P* < 0.05 vs PM group. Different lowercase letters indicate significant differences *P* < 0.05.

As shown in Fig. 6A, 7-day exposure to PM resulted in the disappearance of alveolar septa, disruption of the hexagonal structure of pulmonary lobules, and increased inflammatory cell infiltration in broiler lungs. In the PM+0.4% SP group, H&E staining showed partial restoration of alveolar septa and improvement in lobular structure integrity. Lung injury scores in the PM group were significantly higher than those in the CON group (*P* < 0.05; Fig. 6B). However, no significant differences were observed between the PM group and the PM+0.1%SP or PM+0.2%SP groups (*P* > 0.05). In contrast, the PM+0.4%SP group showed a significantly lower lung injury score compared to the PM group (*P* < 0.05).

Based on lung injury scores, 0.4% SP was found to alleviate PM_2.5_-induced pulmonary damage in broilers. Therefore, subsequent analyses focused primarily on the PM+0.4%SP group. The levels of IFN-γ, IL-12, and IL-18 proteins in lung tissues were significantly elevated in the PM group compared to the CON group (*P* < 0.05; Fig. 6C), whereas these cytokine levels were significantly reduced in the PM+0.4%SP group compared to the PM group (*P* < 0.05). The relative mRNA expression level of *IL-1β* in lung tissues was significantly higher in the PM group than in the CON group (*P* < 0.05) and significantly lower in the PM+0.4%SP group than in the PM group (*P* < 0.05; Fig. 6D). Additionally, the *IL-6* mRNA level was significantly reduced in the PM+0.4%SP group compared to the PM group (*P* < 0.05; Fig. 6D), while no significant difference was observed between the PM and CON groups (*P* > 0.05).

### Sodium propionate on pulmonary immune function in broilers exposed to PM

**Fig 7:**
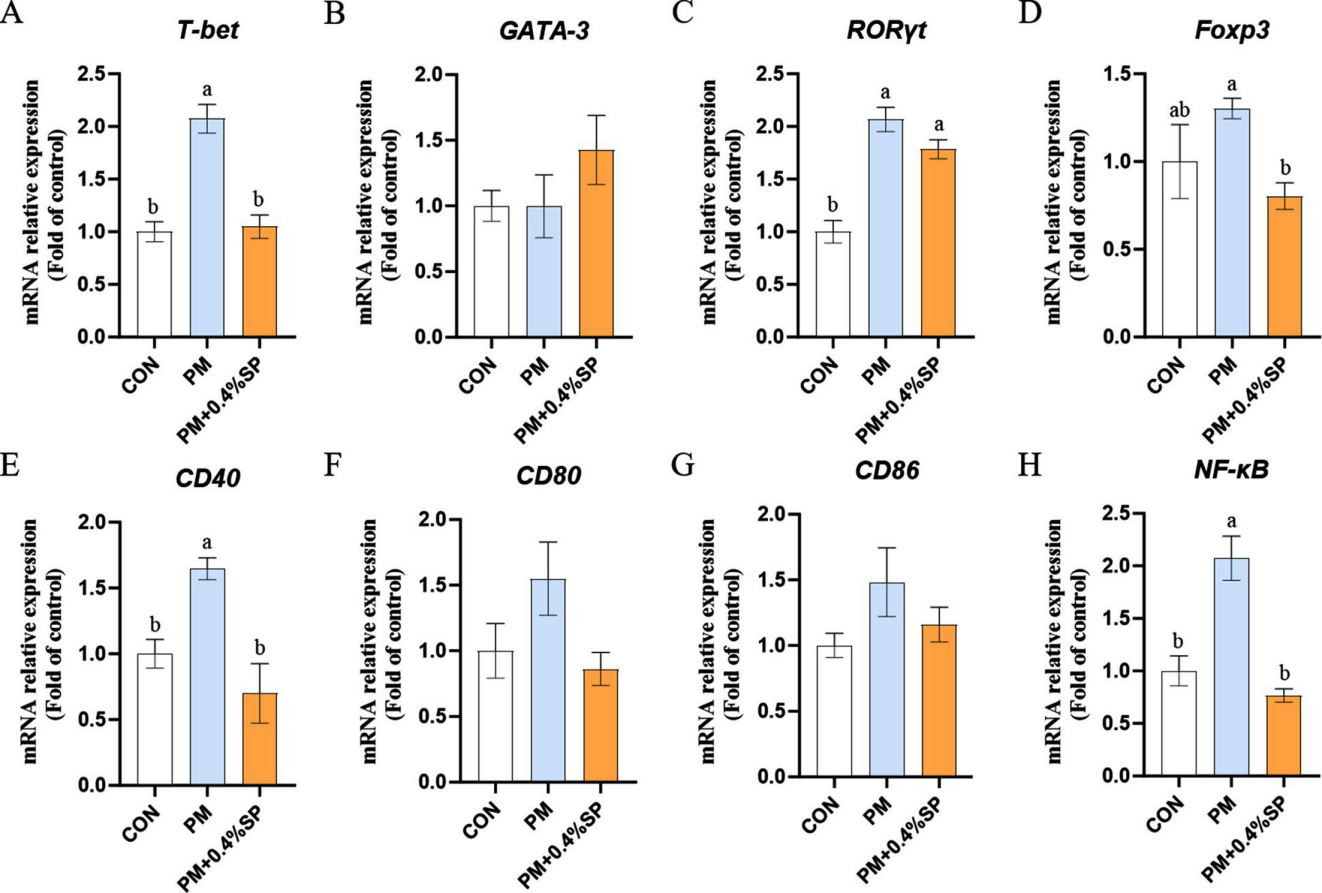
T cell transcription factors and dendritic cell activity gene expression in broilers treated with 0.4% SP after PM exposure. T cell transcription factors (**A–D**) *T-bet, GATA3, RORγt,* and *FOXP3* mRNA expression. Dendritic cell activity (**E–H**) *CD40, CD80, CD86*, *NFκB* mRNA expression. Different lowercase letters indicate significant differences *P* < 0.05. *n* = 8.

**Fig 8:**
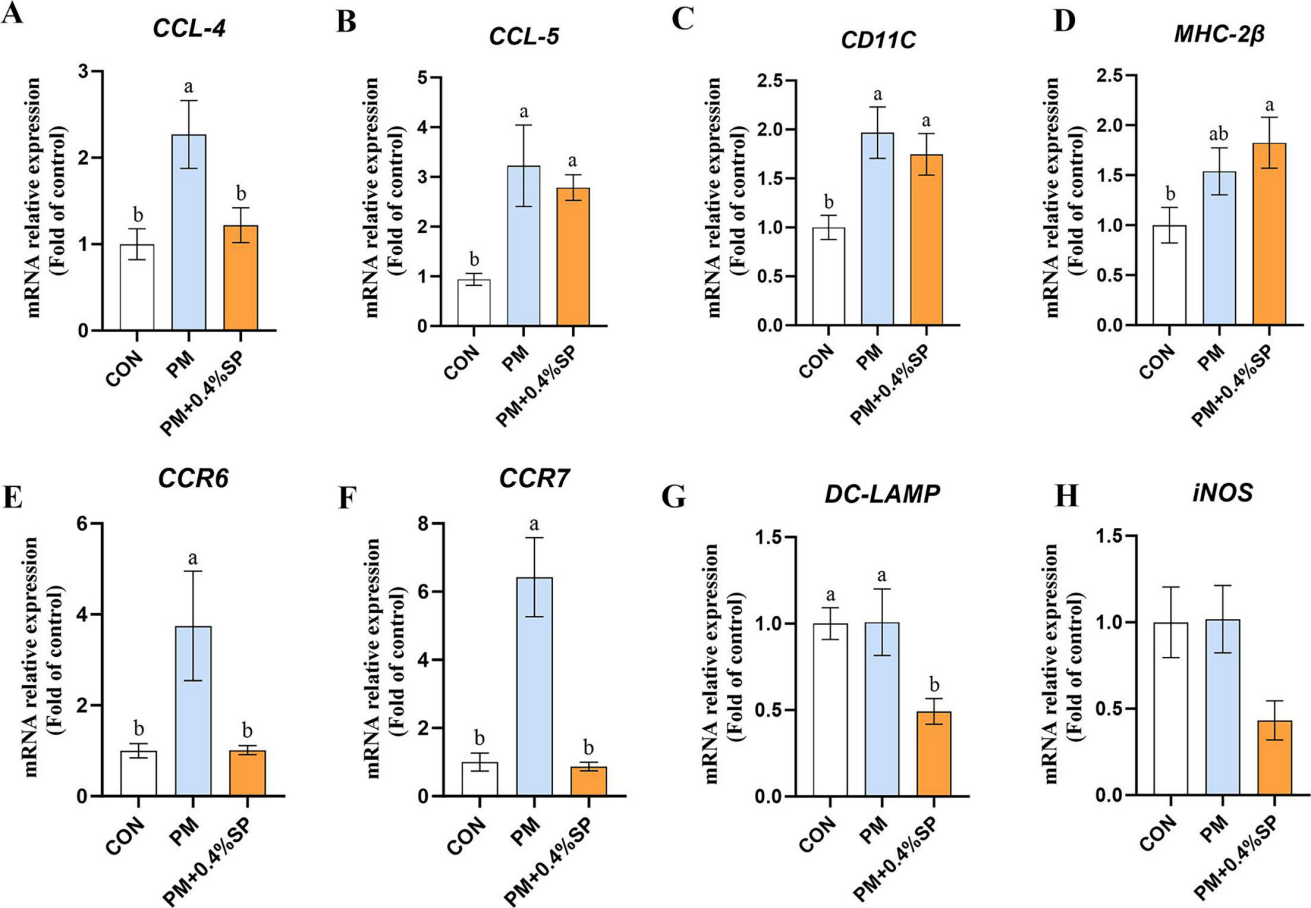
APC cell activity and dendritic cell maturation gene expression in broilers treated with 0.4% SP after PM exposure. APC cell activity (**A–D**) *CCL4, CCL5, CD11C,* and *MHC-IIβ* mRNA expression. Dendritic cell maturation (**E–H**) *CCR6, CCR7, DC-LAMP,* and *iNOS* mRNA expression. Different lowercase letters indicate significant differences *P* < 0.05. *n* = 8.

The mRNA expression levels of *T-bet* and *RORγt* in lung tissues were significantly higher in the PM group than in the CON group (*P* < 0.05; Fig. 7A and C). However, *T-bet* expression in the PM+0.4%SP group was significantly lower than in the PM group (*P* < 0.05), with no significant difference from the CON group. Compared to the CON group, *CD40* mRNA expression in the PM group was significantly upregulated (*P* < 0.05), while the PM+0.4%SP group showed significantly reduced *CD40* expression compared to the PM group (*P* < 0.05; Fig. 7D), with no significant difference from the CON group. The mRNA expression levels of *CCL4, CCL5,* and *CD11C* were significantly higher in the PM group than in the CON group (*P* < 0.05; Fig. 8A through C). In the PM+0.4%SP group, *CCL*4 expression was significantly lower than in the PM group (*P* < 0.05), and not significantly different from the CON group. *CCL5* expression in the PM+0.4%SP group was significantly higher than in the CON group (*P* < 0.05), with no significant difference from the PM group. Similarly, *CD11C* expression in the PM+0.4%SP group was significantly higher than in the CON group (*P* < 0.05), but not significantly different from the PM group. The expression level of *MHC-IIβ* in the PM+0.4%SP group was significantly higher than in the CON group (*P* < 0.05; Fig. 8D) but showed no significant difference compared to the PM group (*P* > 0.05). The mRNA expression levels of *CCR6* and *CCR7* were significantly increased in the PM group compared to the CON group (*P* < 0.05; Fig. 8E and F), and both were significantly reduced in the PM+0.4%SP group compared to the PM group (*P* < 0.05). Notably, *DC-LAMP* expression in the PM+0.4%SP group was significantly lower than in both the CON and PM groups (*P* < 0.05; Fig. 8G).

## DISCUSSION

In this study, we demonstrate that broilers exposed to total suspended particulates (TSP) rich in PM_2.5_ develop pronounced pulmonary injury accompanied by dysbiosis of the cecal microbiota. Histological collapse of the hexagonal lobular architecture and upregulation of IL-1β, IL-2, IL-6, IL-8, and IL-10 confirm that PM_2.5_ provokes acute lung inflammation, consistent with mammalian reports showing PM_2.5_ activation of TLR4/MAPK/NF-κB and NLRP3 inflammasome pathways (23, 24). Importantly, these inflammatory changes coincided with increased gut microbial α-diversity but a skewed community composition—expansion of pro-inflammatory *Alistipes* and *Rikenellaceae*, and depletion of *Parabacteroides* and *B. uniformis*—mirroring gut–lung axis disturbances observed in sepsis and ARDS models (25, 26). Mechanistically, particulate matter that reaches the alveoli carries endotoxins and microbial fragments that bind pattern-recognition receptors on alveolar macrophages (AMs) and epithelial cells, triggering cytokine cascades. The consequent rise in reactive oxygen species and epithelial damage not only impairs gas exchange but also releases alarmins that dysregulate systemic immunity. Our data, showing NF-κB activation and heightened pro-inflammatory cytokine mRNA in lungs, align with this model and underscore the centrality of innate sensors in PM_2.5_-induced pathology.

Beyond local effects, lung inflammation appears to remodel the gut ecosystem via immune-mediated and neurohumoral pathways. Elevated serum cytokines and altered motility in response to pulmonary insult can disturb gut barrier integrity, allowing luminal antigens to shift microbial niches. The bloom of *Alistipes*—a genus linked to pro-inflammatory metabolites in ARDS—and the rise in the Firmicutes/Bacteroidetes ratio likely exacerbate systemic inflammation (27–29). Simultaneously, loss of SCFA-producing taxa such as *B. uniformis* deprives the host of key anti-inflammatory signals that regulate Treg differentiation and NF-κB suppression (30, 31). It is worth noting that in our other study, the use of antibiotics to intervene in gut microbiota and exposure to PM_2.5_ exacerbated inflammation damage due to the absence of *B. uniformis* (32). Thus, gut dysbiosis not only reflects but also amplifies lung injury via the gut–lung axis. The restoration of *B. uniformis* through oral gavage effectively reversed both gut and lung pathology. By replenishing its population, cecal acetate and propionate levels rebounded, coinciding with reduced lung injury scores, lower expression of APC activation markers (CD40, CCL4), and a shift in T cell polarization away from Th1 (decreased T-bet, IFN-γ, IL-18) toward regulatory phenotypes (increased FoxP3). This dual modulation of innate and adaptive arms underscores *B. uniformis*’s capacity to reestablish mucosal homeostasis: its polysaccharide A engages TLR2 on dendritic cells to induce IL-10, while SCFAs act directly on bone-marrow precursors to generate tolerogenic DCs and Tregs (33, 34).

To isolate the contribution of SCFAs, we supplemented diets with sodium propionate. At 0.4%, propionate attenuated lung injury and dampened DC activation and Th1 skewing but did not promote Treg expansion, implying that additional bacterial signals (e.g., polysaccharide A) are necessary for full regulatory reprogramming. Nevertheless, propionate’s ability to inhibit NLRP3 and NF-κB in macrophages (35, 36) highlights its therapeutic potential as a metabolite-based intervention. These findings suggest that sodium propionate alone, although effective in dampening innate and Th1-driven inflammation, may be insufficient to trigger full Treg induction due to the lack of microbe-associated molecular patterns (MAMPs) derived from commensal bacteria. SCFAs mainly function as epigenetic and metabolic co-factors that enhance Treg differentiation only in the presence of antigen-specific or microbial co-stimulatory cues. In contrast, polysaccharide A (PSA) or other Bacteroides-derived surface glycans can directly engage TLR2 or other antigen-presenting cell pathways to drive tolerogenic DC programming and FoxP3 induction. Therefore, a synergistic interaction between microbial metabolites and structural components, rather than SCFAs alone, may be required to achieve complete immunoregulatory conversion. Future work should explore combined supplementation strategies (e.g., live Bacteroides, purified PSA, or postbiotic complexes) to validate this cooperative model. Notably, PM_2.5_ exposure did not significantly affect broiler growth performance in our setting in contrast to prior work attributing early growth retardation to PM components (5, 7). This discrepancy may reflect differences in background rearing conditions, stress from gavage procedures, or the relatively short exposure window. Nevertheless, propionate tended to improve feed conversion ratio, suggesting benefit under subclinical challenge.

Nevertheless, this work has limitations. The use of TSP rather than isolated PM_2.5_ may confound particle size-specific effects. Our short-term exposure model captures acute inflammation but not chronic adaptation. Future studies should employ real-world PM_2.5_ generators, longer exposures, and challenge with respiratory pathogens to gage functional resistance. Detailed profiling of dendritic-T cell synapses, single-cell transcriptomics of lung and gut immune cells, and metabolomic mapping will deepen mechanistic insight. Moreover, field trials assessing productivity and disease incidence under probiotic or SCFA-enhanced regimens will be critical for translation.

### Conclusion

In conclusion, PM_2.5_ exposure provokes lung inflammation and gut dysbiosis in broilers through interconnected innate and adaptive pathways. The restoration of *B. uniformis* or its key metabolite propionate rebalances the gut–lung axis, suppresses APC overactivation, and mitigates Th1-driven inflammation. These data provide a scientific basis for microbiota- and metabolite-based strategies to safeguard poultry respiratory health in polluted environments.

## Data Availability

Raw metagenome sequencing data are available in the SRA database under accession number PRJNA979789.

## SUPPLEMENTAL MATERIAL

Supplemental material aem.01841-25-s0001.docx Figures S1 to S3.

Graphical abstract aem.01841-25-s0002.tif Visual depiction of the study.

ASM does not own the copyrights to Supplemental Material that may be linked to, or accessed through, an article. The authors have granted ASM a non-exclusive, world-wide license to publish the Supplemental Material files. Please contact the corresponding author directly for reuse.
