# Supplementary material for: Simulated poultry-house PM2.5 exposure reveals a gut–lung axis mechanism of microbial propionate in protecting against pneumonia
Source: Appl Environ Microbiol. 2026 Jan 14;92(2):e01841-25. doi: 10.1128/aem.01841-25 (PMC12915341; doi:10.1128/aem.01841-25)
Supplement: Supplemental material — Figures S1 to S3. [file aem.01841-25-s0001.docx]

Figure S1 Work flow of three trials


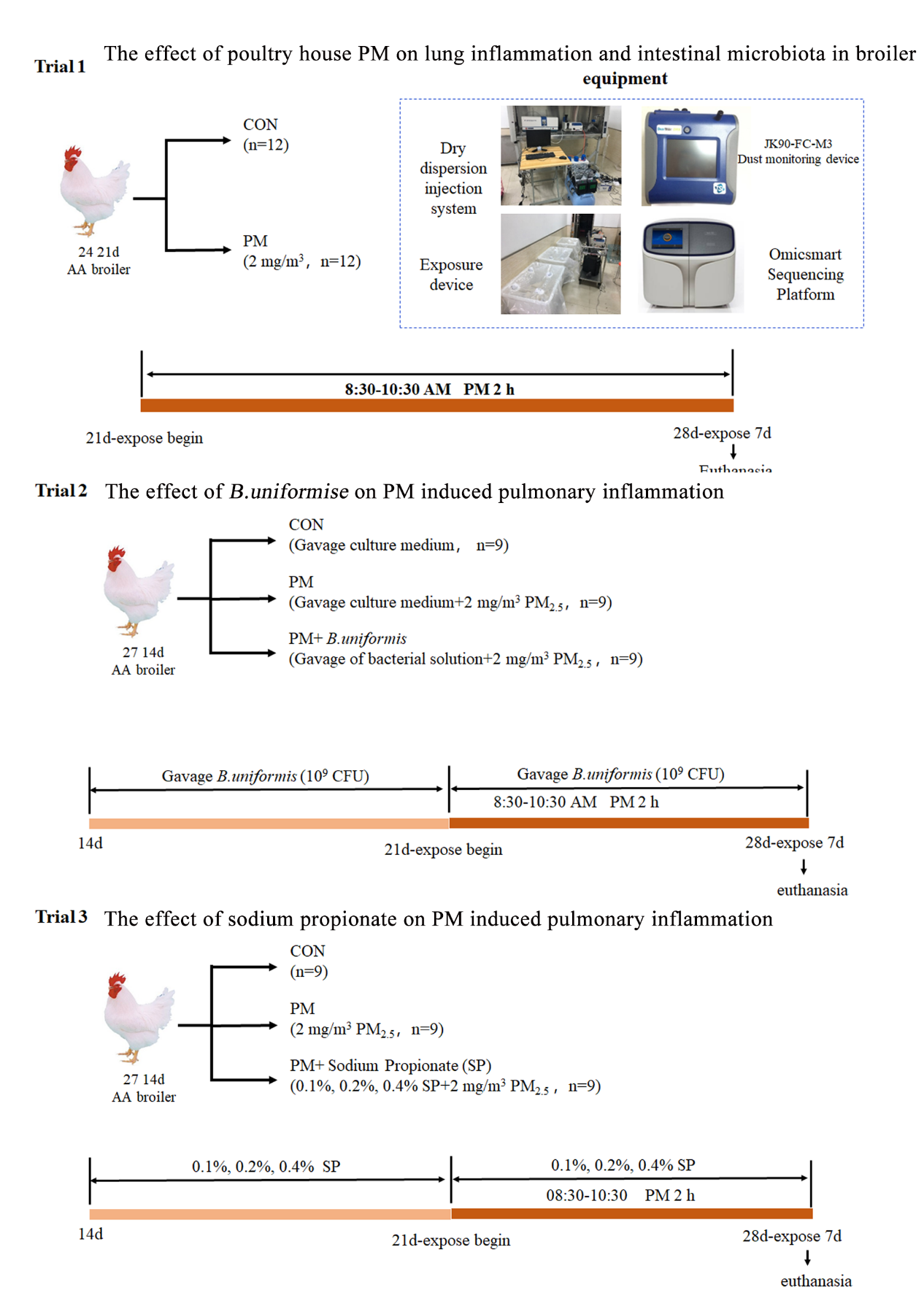


Figure S2 Actual exposure concentration of particles of different sizes


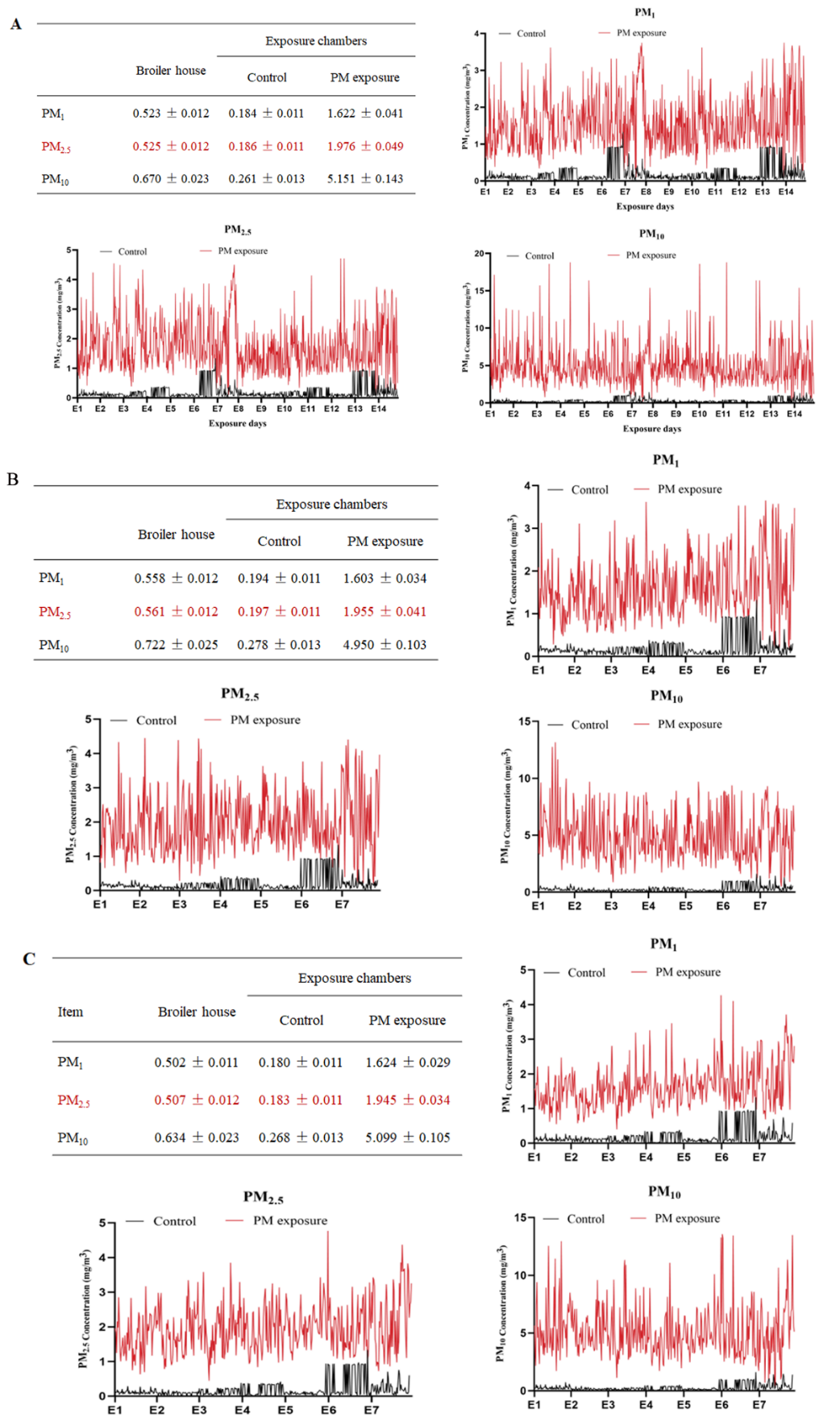


Note: A-C: Actual exposure concentration of particles of different sizes of trial 1-3

Figure S3 The rarefaction depth of 16S rRNA sequencing


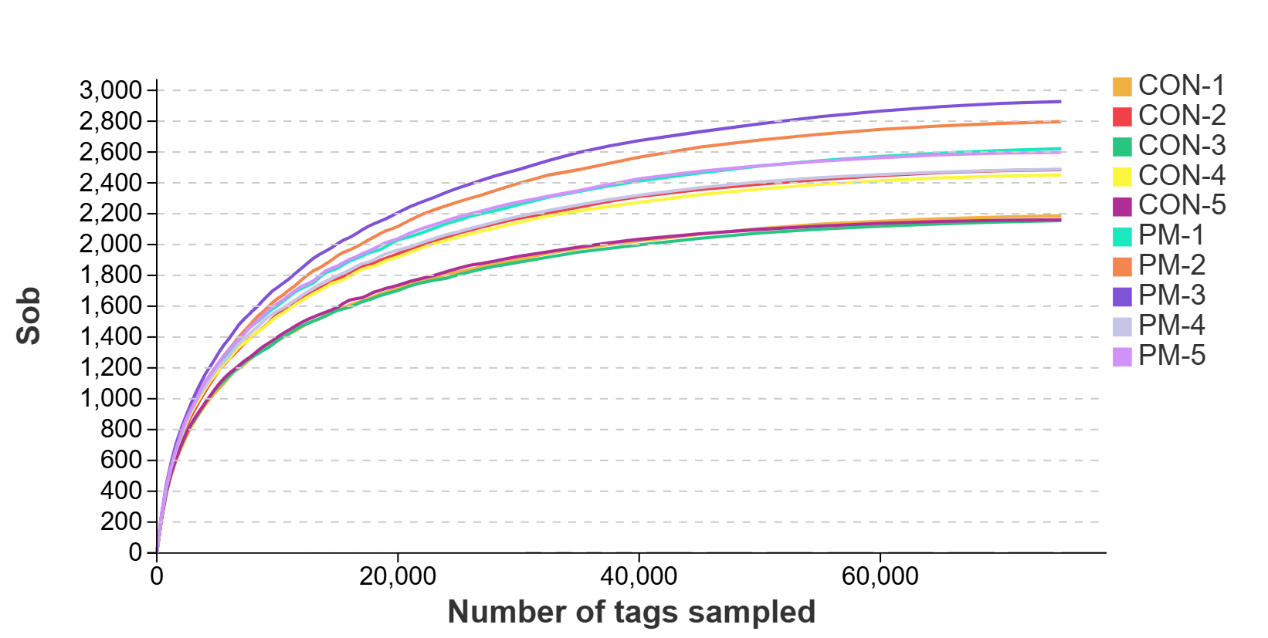


Note: n=5 per group.
