# Supplementary figures and images for: Simulated poultry-house PM2.5 exposure reveals a gut–lung axis mechanism of microbial propionate in protecting against pneumonia
Source: Appl Environ Microbiol. 2026 Jan 14;92(2):e01841-25. doi: 10.1128/aem.01841-25 (PMC12915341; doi:10.1128/aem.01841-25)

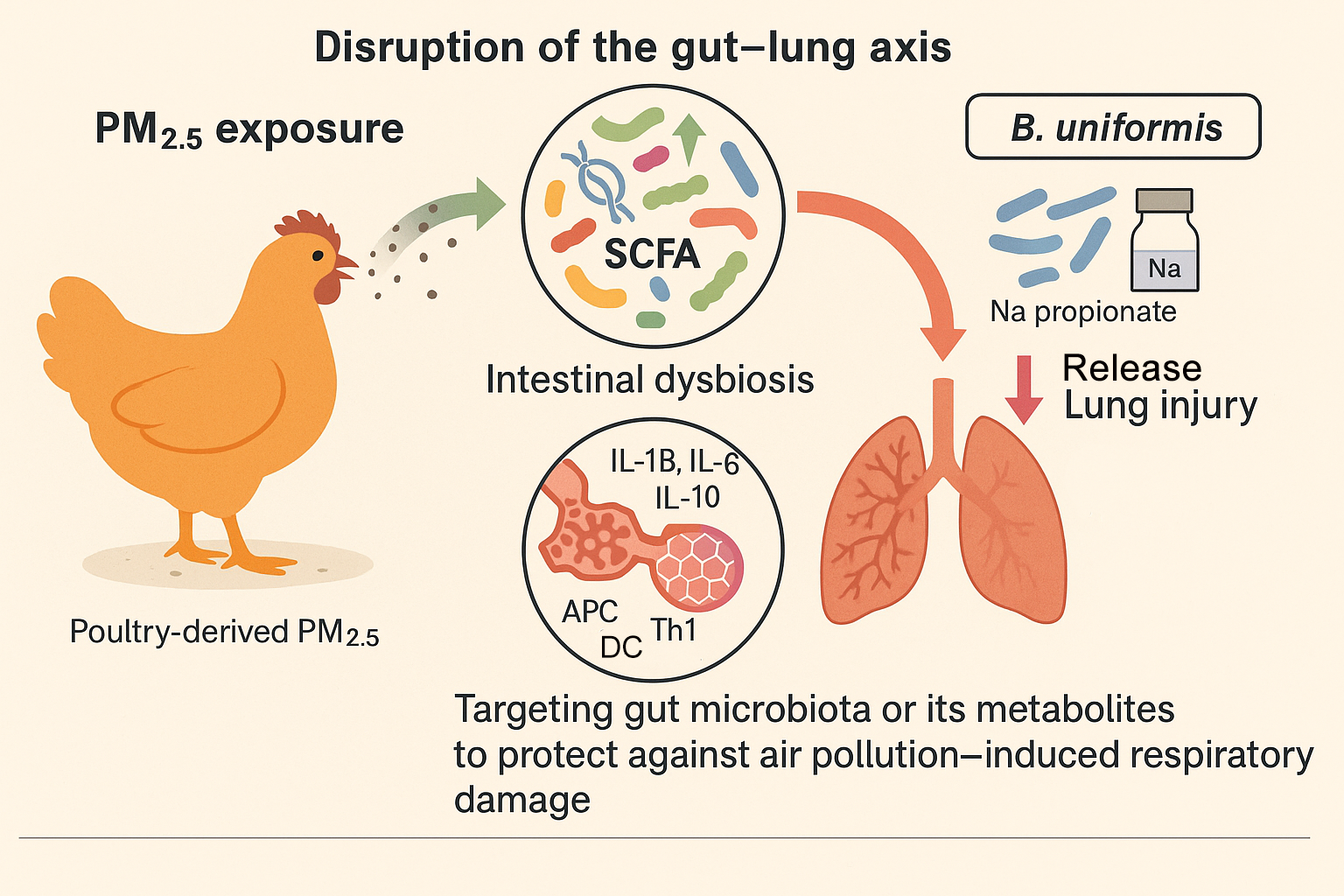

Supplement: Graphical abstract — Visual depiction of the study. [file aem.01841-25-s0002.tif]
